# Metabolomic based approach to identify biomarkers of broccoli intake†

**DOI:** 10.1039/d2fo03988e

**Published:** 2023-08-29

**Authors:** Aoife E. McNamara, Xiaofei Yin, Cassandra Collins, Lorraine Brennan

**Affiliations:** a UCD School of Agriculture and Food Science, Institute of Food and Health, University College Dublin Belfield Dublin 4 Ireland lorraine.brennan@ucd.ie +353 1 7166815; b UCD Conway Institute of Biomolecular and Biomedical Research, University College Dublin Dublin Ireland

## Abstract

It is well-established that consumption of cruciferous and brassica vegetables has a correlation with reduced rates of many negative health outcomes. There is an increased interest in identifying food intake biomarkers to address limitations related to self-reported dietary assessment. The study aims to identify biomarkers of broccoli intake using metabolomic approaches, examine the dose–response relationship, and predict the intake by multimarker panel. Eighteen volunteers consumed cooked broccoli in A-Diet Discovery study and fasting and postprandial urine samples were collected at 2, 4 and 24 hours. Subsequently the A-Diet Dose–response study was performed where volunteers consumed different portions of broccoli (49, 101 or 153 g) and urine samples were collected at the end of each intervention week. Urine samples were analysed by ^1^H-NMR and LC-MS. Multivariate data analysis and one-way ANOVA were performed to identify discriminating biomarkers. A panel of putative biomarkers was examined for its ability to predict intake through a multiMarker model. Multivariate analysis revealed discriminatory spectral regions between fasting and fed metabolic profiles. Subsequent time-series plots revealed multiple features increased in concentration following the consumption. Urinary *S*-methyl cysteine sulfoxide (SMCSO) increased as broccoli intake increased (0.17–0.24 μM per mOSM per kg, *p* < 0.001). Similarly from LC-MS data genipin, dihydro-β-tubaic acid and sinapic acid increased with increasing portions of intake. A panel of 8 features displayed good ability to predict intake from biomarker data only. In conclusion, urinary SMCSO and several LC-MS features appeared as potentially promising biomarkers of broccoli consumption and demonstrated dose–response relationship. Future work should focus on validating these compounds as food intake biomarkers.

## Abstract 2

The metabolomic-based approaches were applied to identify biomarkers of broccoli intake, which demonstrated a good dose–response relationship and indicated promising potential for predicting intake.

## Graphical abstract

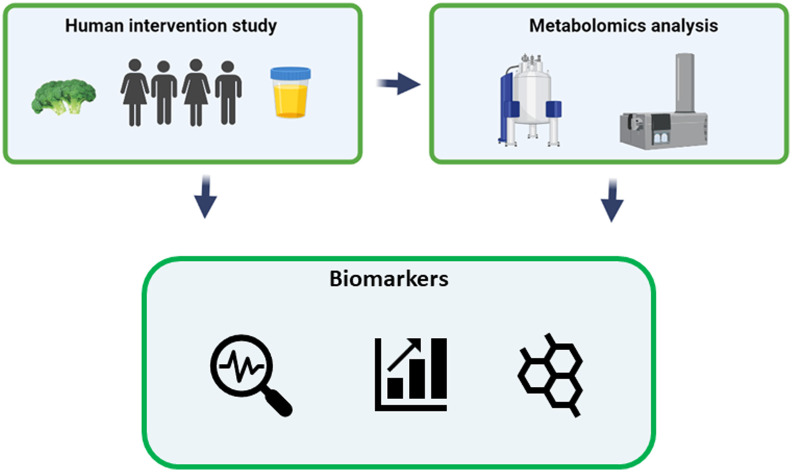

## 1 Introduction

Broccoli is a member of a group known as cruciferous or brassica vegetables which also includes kale, cabbage, Brussels sprouts, cauliflower and others.^1^ Brassica are among the most important vegetables in Europe as they are a popular food, cheap and easily accessible.^2^ Brassica are rich in beneficial plant metabolites and many of the associated health benefits are linked to their glucosinolate-derived phytochemical content.^3^ A recent meta-analysis found cruciferous consumption was inversely associated with stroke, cardiovascular disease (CVD), cancer and all-cause mortality, with greatest effects at intakes >200 g day^−1^.^4^ However, levels of heterogeneity between studies were moderate to high for stroke, cardiovascular disease and total cancer and high for all-cause mortality. One of the factors contributing to the inconsistent results could be the difficulty in obtaining accurate dietary exposure data.

Traditional commonly used dietary assessment techniques are self-reported methods which are subject to well documented limitations such as inaccurate portion size estimation, recall bias and misreporting.^5–8^ These limitations highlight the need to develop more accurate and objective dietary assessment measures, for example, the identification of food intake biomarkers. Food intake biomarkers are single metabolites, or a combination of metabolites, reflecting the consumption of either a specific food or food group, displaying a clear time- and dose–response after intake.^9^

Metabolomics, the study of small metabolites, plays a key role in the discovery of food intake biomarkers. Multiple studies examining cruciferous vegetable intake assessment are present in literature.^10–15^ The most promising biomarkers of cruciferous vegetables discovered to date are sulphur-containing compounds such as: sulforaphane,^10,11^ SMCSO (*S*-methylcysteine sulfoxide)^12^ and other compounds from the isothiocyanate class.^10,11,15^ Sulforaphane, an isocyanate, is abundantly present in cruciferous vegetables,^16^ and sulforaphane *N*-acetylcysteine and sulforaphane *N*-cysteine were identified by Andersen *et al.*, (2013)^11^ as urinary markers of brassica containing meals in a cross-over meal study representative of the new Nordic diet. Upon further analysis, sulforaphane *N*-acetylcysteine was discovered to be a strong brassica intake biomarker (sensitivity and specificity >80%). SMCSO and 3 of its metabolites were identified as urinary biomarkers of cruciferous intake following a high cruciferous intake dietary intervention (500 g d^−1^ of broccoli and Brussels sprouts for 14 days).^12^ A range of acetyl-cysteine derivatives of isothiocyanates have been identified as biomarkers of brassica-containing diets including *N*-Acetyl-(*N*′-benzylthiocarbamoyl)-cysteine, Iberin *N*-acetyl cysteine,^10,11^ and iberin.^15^ However there has been no studies attempting to validate the compounds as broccoli biomarkers or to assess their ability to predict intake. To this end the objectives of the present study were as follows: (1) to use a metabolomics approach to identify and confirm biomarkers related to broccoli consumption; (2) to examine the dose–response relationship and (3) to assess the ability of a panel of potential biomarkers for predicting food intake.

## 2 Materials and methods

### 2.1 A-diet discovery study

Ethical approval for the A-Diet Discovery study was granted by the UCD Sciences Human Research Ethics Committee (LS-15-69-Brennan). The study design has been previously been described in detail,^17^ to identify novel biomarkers of 9 commonly consumed foods (apples, broccoli, peppers, oranges, white bread, wholemeal bread, spaghetti, cheese and Madeira cake.). The primary test food of interest for this research was broccoli and apple intake was used as a control food and we reported only the data with respect to the discovery of broccoli biomarkers. The inclusion criteria included healthy, non-pregnant/lactating, non-smokers, an age range between 18 and 60 years old, and a body mass index (BMI) between 18.5 and 30 kg m^−2^. Exclusion criteria included any diagnosed health condition (chronic or infectious diseases), consumption of medications/nutritional supplements or any allergies/intolerances to the test foods. Furthermore, participants were asked to avoid consuming alcohol, medication, and foods related to specific food (apples or broccoli) for 24 h prior to biofluid collection. Participants consumed 135 g of cooked broccoli and 360 g of raw apples, and provided fasting first void and postprandial (2 h and 4 h) urine samples. Following 24 h a fasting first void urine sample was collected.

### 2.2 A-diet dose–response study

Ethical approval for the A-Diet Dose–response study was granted by the UCD Sciences Human Research Ethics Committee (LS-17-16-Brennan). Inclusion criteria for the dose–response study were the same as for the discovery study above. Details of the study design are published elsewhere.^17^ Participants were assigned to either a lunch (*N* = 27) or dinner (*N* = 34) test meal group and invited to partake in a 5-week study. Each week the portion sizes of each test food changed (high, medium or low portion). Participants received the meals in random order. The high, medium and low broccoli portions were 153 g, 101 g, and 49 g and apple portions were 300 g, 100 g and 50 g, respectively. During the test week, participants were also asked to avoid consuming any other foods related to the test meal ingredients.

Fasting first void urine was collected after an overnight 12 h fast at the end of each test week after participants had consumed the test meal for 4 days and chilled until processing. All urine samples were centrifuged at 1800*g* for 10 min at 4 °C. Aliquots of 1 mL were stored at −80 °C for later analysis.

### 2.3 Metabolomic analysis of urine samples

The ^1^H nuclear magnetic resonance (^1^H-NMR) spectroscopy and liquid chromatography mass spectrometry (LC-MS) based techniques were applied for urine metabolomic analysis. Urine samples from participants were prepared for ^1^H-NMR spectroscopy analysis first defrosted and then prepared by addition of 250 μL phosphate buffer (0.2 mol KH_2_PO_4_ per L, 0.8 mol K_2_HPO_4_ per L) to 500 μL urine, and the mixed sample was then centrifuged at 5360*g* for 5 min at 4 °C. The collected supernatant (540 μL) were mixed with10 μL sodium trimethylsilyl [2,2,3,3-2H_4_] proprionate (TSP) and 50 μL deuterium oxide (D_2_O). Subsequently, the samples were transferred into NMR tubes for analysis. Spectra were acquired on a 600 MHz Varian Spectrometer (Varian Limited, Oxford, United Kingdom) by using the first increment of a nuclear Overhauser enhancement spectroscopy pulse sequence at 25 °C. A total of 16 384 data points and 128 scans was used, and water suppression was achieved during the 2.5 s of relaxation delay and the 100 ms of mixing time. All ^1^H-NMR urine spectra were referenced to TSP at 0.0 parts per million (ppm) and then processed manually in the Chenomx NMR Suite software (version 7.7) by using a line broadening of 0.2 Hz, followed by phase and baseline correction. Data were normalized to the total area of the spectra integral. NMR spectra from the Discovery study were exported at high resolution using 7500 spectral regions, furthermore the water region was excluded. The exported spectral region dataset was used for multivariate data analysis to extract the most discriminated molecule features. Metabolites were identified using the Chenomx library. To confirm the correct peak assignment of metabolites a pure standard of SMCSO was prepared and analysed by ^1^H-NMR. A 50 μL solution of pure standard and phosphate buffer (0.0025 mol L^−1^) was added to a urine sample. The ^1^H-NMR spectra were acquired prior to and after the addition of the pure compound using the same parameters as study samples. The spectra of the pure SMCSO standard, the urine and urine spiked with SMCSO were compared to confirm the identification of SMCSO. Osmolality was measured using an Advanced Micro Osmometer model 3300 (Advanced Instruments) employing freezing point depression. Values are reported as the number of solute particles, in moles, dissolved in a kilogram of urine (mOsm per kg). Profiled urinary metabolite concentrations from NMR analysis were normalized to osmolality.

Urine samples were analysed by LC-MS as previously described.^18^ A mixture of five isotope labelled compounds (malic acid d_3_, methionine d_3_, myristic acid ^13^C, adipic acid d_4_ and succinic acid d_4_, each at 10 μg mL^−1^ in 20% (v/v) EtOH/Millipore H_2_O) as internal standards were used. Test urine and pooled urine samples (used as Quality Control (QC) samples) were thawed on a roller for 30 min and centrifuged at 1800*g* for 5 min at 4 °C. Urine samples (100 μL) were mixed with 100 μL internal standards, and then vortexed at 35 Hz for 10 s and centrifuged at 2000*g* for 2 min. The supernatant was subsequently transferred to LC-MS vials with 250 μL inserts and placed into LC autosampler at 4 °C.

An Agilent LC-QTOF-MS, consisting of a 1290 Infinity II LC system and an Agilent Jetstream Electrospray ionization (ESI) source coupled to a 6545 QTOF mass spectrometer were applied to perform urine metabolomic analysis. The chromatography was performed in reverse phase mode with a Zorbax eclipse plus C18 column (2.1 × 50 mm, 1.8 μm) coupled to a Zorbax eclipse plus C18 guard column (2.1 × 5 mm, 1.8 μm). The column temperature was set as 30 °C, and injection volume was 5 μL. The mobile phase A and B were water and 80% of acetonitrile, respectively, both with 0.1% formic acid. The flow rate was 0.4 mL min^−1^, and the gradient conditions were set as follows: 1% B (0–1.5 min), 11% B (1.5–9 min), 25% B (9–15 min), 50% B (15–18 min), 99% B (18–18.05 min), 99% B (18.05–21 min), 1% B (21–21.05 min) and 1% B (21.05–23 min). The MS parameters used for the analysis: drying gas temperature, 325 °C; drying gas flow rate, 10 L min^−1^; sheath gas temperature, 350 °C; sheath gas flow rate, 11 L min^−1^; nebulizer pressure, 45 gauge pressure (pounds per square inch); capillary voltage, 3500 V; nozzle voltage, 1000 V; fragment or voltage, 100 V; skimmer, 45 V. The mass range of mass-to-charge-ratio (*m*/*z*) was between 50 and 1600, and the 2 GHz extended dynamic range mode was used. Application of centroid mode at scan rate of 1 spectra per s was used to collect data. Samples were run by using both positive and negative ionization modes.

Data were acquired using MassHunter acquisition B.08.00 software (B.08.00.8058.3 Sp1 Agilent Technologies) and were further processed in MassHunter qualitative analysis software (B.07.00 Sp2 Agilent Technologies). Molecular features were extracted using the molecular feature extractor (MFE) algorithm, and a list of features were generated with retention time, *m*/*z*, adducts or isotopes of compounds, signal intensity and accurate mass. The target data type was set to small molecules and the threshold value for peak height was set to 5000 counts. The isotopic peak spacing tolerance was 0.0025 *m*/*z* with a maximum charge state of 1 and isotope model was set to common organic molecules. The Compound Exchange Format (.cef) files, transformed from the original spectral data, were generated and imported into MassProfiler (Version B.07.01; build 99.0 Agilent Technologies) in order to align compounds and filter data. The alignment parameters were set with a retention time tolerance ±0.3 min and a mass tolerance of ±15 ppm + 2.0 mDa. The final dataset was exported including molecule features with retention time, mass and feature abundance. For features with missing values, they were replaced with half the minimum abundance value for that particular feature. Feature abundances were normalised to osmolality before performing multivariate data analysis.

The interesting features were initially identified in MassHunter ID Browser (B 7.0.799.2 Agilent Technologies) by putative formula generation and an accurate mass data search against a local METLIN (METabolite LINk, https://metlin.scripps.edu) database. Tandem MS (MS/MS) with 3 collision energies 10 eV, 20 eV and 40 eV under the same chromatographic conditions were further performed to attain structural confidence for the metabolites identified by the accurate mass. MS/MS compound identification efforts included fragmentation modelling using CFM ID^19^ and fragment matches with the Human Metabolome Database (HMDB).^20^ Fragment data generated from MS/MS was also input into METLIN MS/MS Spectrum Match Search (precursor *m*/*z* and top 30 most intense fragments *m*/*z*) in order to confirm identification. Furthermore, some authentic standards were analysed by the same LC-MS/MS method to confirm the identification. All metabolites were attributed to one of four levels of confidence in metabolite identification according to Metabolomics standards initiative: Level I for identified compounds; Level II for putatively annotated compounds; Level III for putatively characterized compound classes; Level IV for unknowns.^21^

### 2.4 Statistical analysis

Data from NMR and LC-MS from the A-Diet Discovery study were analysed using Multivariate Empirical Bayes Analysis of Variance (MEBA) for Time Series in Metaboanalyst (https://www.metaboanalyst.ca/). Features with significant changes over time (using the Hotelling-T2 values) were examined for their dose–response in the A-Diet Dose–response study. One-way repeated measures analysis of variance (ANOVA) was performed on the dose–response data. A *p* value < 0.05 was considered to indicate significance.

Using the multiMarker web application a panel of putative biomarkers was examined for its ability to predict intake.^22^ The multiMarker approach is a factor analytic model that expresses the relationship between the biomarkers and the latent intake. The model is built within a Bayesian framework which allows estimation of uncertainties in the predicted intake. The training set was established from randomly selecting 90% of the data to build the model and the rest of data were used to predict intake using biomarker data. This was repeated in an iterative fashion to investigate the agreement between the predicted and actual intake.

## 3 Results

### 3.1 Identification of biomarkers of broccoli intake

In total, 8 males and 12 females were recruited for the Discovery Study. One participant attended for baseline measurements only. From the remaining volunteers, 18 completed the broccoli test visit (ESI Fig. 1†). The participants’ mean age was 34 years old, with a mean body mass index (BMI) of 23.88 kg m^−2^ (Table 1).

**Table 1:** Demographics for A-Diet Discovery and Dose–response studies for broccoli intake

Characteristics	Discovery study (*N* = 18)	Dose–response study (*N* = 32)
Gender	8M 10F	16M 16F
Age (y)	34 ± 12	29 ± 10
Anthropometrics		
BMI (kg m^−2^)	23.84 ± 2.98	23.96 ± 2.99
W : H	0.77 ± 0.07	0.84 ± 0.06

After conducting MEBA for Time Series on ^1^H-NMR data, the top 30 spectral regions were selected and examined against the control food at 0, 2, and 4 h time points. The analysis revealed multiple spectral regions which exhibited an increased peak intensity with time following broccoli consumption only (ESI Fig. 2†) and these spectral regions were listed in Table 2 with their potential identification.

**Table 2:** Spectral regions of interest and their potential identifications

Spectral region (ppm)^a^	Hotelling-T2	Potential identification
2.7675	39.066	Levulinate
2.7685	30.431	Levulinate
1.3455	27.932	2-Hydroxyisobutyrate
2.7925	27.835	Unknown 1
2.7935	27.657	Unknown 1
2.7695	25.597	Levulinate
2.7395	24.35	Sarcosine
3.0365	23.95	Creatinine
2.8195	22.839	SMCSO
1.8925	22.575	Acetate
1.8915	22.125	Acetate
2.8205	21.969	SMCSO

a A selection of top-ranking regions from MEBA timecourse analysis are presented. These regions represent peaks and one metabolite can have multiple peaks depending on the chemical structure of the metabolite. ppm, parts per million; SMCSO, *s*-methyl cysteine sulfoxide.

**Fig. 1:**
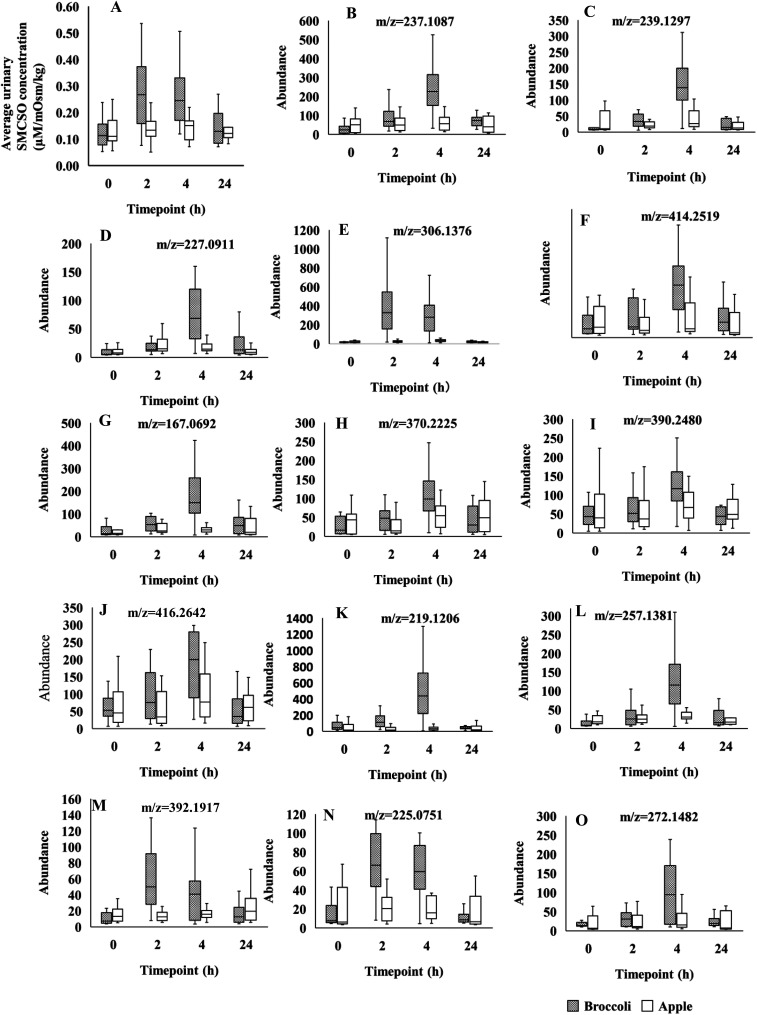
(A) Box plots of *S*-methyl cysteine sulfoxide (SMCSO) concentrations in NMR analysis across the 4 time-points (0, 2, 4, 24 h), following consumption of broccoli and apples in separate test sessions in A-Diet Discovery study. (B–O) Box plots of fourteen LC-MS feature intensities across the 4 time-points (0, 2, 4, 24 h). Grey and white boxes represent broccoli and apple group, respectively. The solid black line denotes the median of the group. Figure titles indicated the *m*/*z* of features. Those features were identified in positive mode.

A number of these spectral regions of interest were assigned to SMCSO and identification confirmed through comparison with an authentic analytical standard (ESI Fig. 3†). Other regions were identified as acetate, levulinate, 2-hydroxyisobutyrate, creatinine and sarcosine (Table 2). Profiling of urinary SMCSO revealed that there was a significant increase in the postprandial samples at 2 and 4 hours compared to fasting and 24 hours (Fig. 1A). After 24 hours, the level of SMCSO was back to the similar level as 0 hour. On the contrary, the level of SMCSO after apple intake remained the same across 4 time points.

**Fig. 2:**
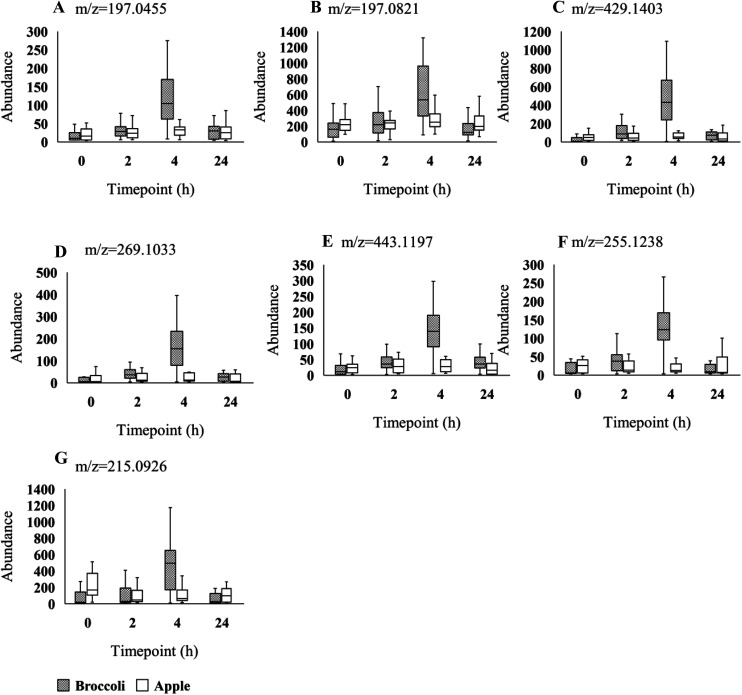
Box plots of seven LC-MS feature intensities (A-G) across the 4 time-points (0, 2, 4, 24 h). Grey and white boxes represent broccoli and apple group, respectively. The solid black line denotes the median of the group. Figure titles indicated the m/z of features. Those features were identified in negative mode.

Using the LC-MS data a total of 1741 features were obtained from urine samples in positive mode and 2086 features were obtained in negative mode. Following MEBA analysis the top 30 features were selected and examined for increases following broccoli consumption compared to control food. A total of 14 features in positive and 7 features in negative indicated an increase after the consumption and also displayed discriminating time profiles when compared to the control food. The results displayed in Fig. 1B–O and Fig. 2A–G show the increased intensity following broccoli intake, whereas the peak intensities didn't change following consumption of the control food, apple.

A molecular formula for each feature was generated by MassHunter using single MS accurate mass data for the molecular ion and its isotopes. Fourteen features of interest in positive, and seven in negative mode, were selected for LC-MS/MS for more complete metabolite identification (Table 3). Matching of MS/MS fragments between urine sample and standard confirmed the identification of Sinapic acid (ESI Fig. 4†). The identification of 3-hydroxy-sebacic acid (*m*/*z* 219.1206) was based on comparison with an authentic standard of the functional parent compound Sebacic acid. The fragmentation pattern obtained from the standard reflected key fragments from the urine sample with a spectral shift of 2 Da (*m*/*z* 97.1010 and 95.0853, 121.1012 and 119.0854, 139.1119 and 137. 0960). The difference in mass between sebacic acid (∼202 Da) and 3-hydroxysebacic acid (∼218 Da) is ∼16 Da which corresponds to the mass of the addition of a hydroxy group (–OH) and loss of a double bond (ESI Fig. 5†). Matching of MS/MS fragments between urine sample and standard confirmed the identification of Genipin: the main fragment in the standard was *m*/*z* 149.0595 however in the urine sample *m*/*z* 167.0703 was a prominent fragment, which would indicate addition of a hydroxy group (ESI Fig. 6†).

**Table 3:** LC-MS/MS of compounds of interest

RT	Mass	Prec. *m*/*z*	Ion	MS/MS	Hotelling-T2	Putative name	Putative formula
14.2	236.1048	237.1087	[M + H]^+^	134.0961, 133.0645, 197.1283, 84.0802, 43.0173	47.14	Dihydro-beta-tubaic Acid^c^^,^^f^	C_13_H_16_O_4_
14.4	238.1215	239.1297	[M + H]^+^	69.0695, 134.0961, 57.0699, 123.0438, 43.0535	44.826	Unidentified^a^^,^^g^	C_16_H_14_O_2_
16.4	226.0844	227.0911	[M + H]^+^	167.0703, 121.0645, 168.0736, 122.0678, 85.0283	40.61	Genipin^d^^,^^e^	C_11_H_14_O_5_
14.9	305.1300	306.1376	[M + H]^+^	149.096, 122.0267, 167.1059, 185.118, 85.0278	37.144	Unidentified^a^^,^^g^	C_14_H_19_N_5_OS
16.6	413.2418	414.2519	[M + H]^+^	414.2482, 416.3141, 416.2666, 415.2508, 223.1690	36.066	Unidentified^a^^,^^g^	C_20_H_29_N_8_O_2_
15.8	166.0627	167.0692	[M + H]^+^		35.47	3-(4-Hydroxyphenyl)propionic acid^a^	C_9_H_10_O_3_
16.9	369.2152	370.2225	[M + H]^+^	370.2219, 85.0282, 191.1060, 374.252, 372.2363	35.321	Unidentified^a^^,^^g^	C_18_H_15_N_8_O
15.3	389.2411	390.2480	[M + H]^+^	113.0611, 67.0546, 287.9454, 43.0173, 114.0670	34.664	Unidentified^a^^,^^g^	C_19_H_35_NO_7_
17.5	415.2567	416.2642	[M + H]^+^		34.55	Unidentified^a^	C_21_ H_37_NO_7_
11.2	218.1156	219.1206	[M + H]^+^	119.0854, 137.0960, 95.0853, 109.1014, 91.0542	34.416	3-Hydroxy-sebacic acid^b^^,^^d^^,^^e^	C_10_H_18_O_5_
16.0	256.1311	257.1381	[M + H]^+^		34.142	1-Methoxy-1-(2,4,5-trimethoxyphenyl)-2-propanol^a^	C_13_H_20_O_5_
18.0	391.1852	392.1917	[M + H]^+^		33.728	Orysastrobin	C_18_H_25_N_5_O_5_
9.5	224.0688	225.0751	[M + H]^+^	55.0537, 69.0692, 147.0444, 57.0686, 119.0487	33.706	Sinapic acid^c^^,^^d^^,^^e^	C_11_H_12_O_5_
14.2	271.1427	272.1482	[M + H]^+^	55.0539, 60.0806, 69.0342, 69.0688, 83.0845	28.647	Unidentified^a^^,^^g^	C_14_ H_25_NS_2_
11.8	198.0532	197.0455	[M − H]^−^		50.044	Ethyl gallate^a^	C_9_ H_10_O_5_
15.1	198.0892	197.0821	[M − H]^−^		41.914	Guaifenesin^a^	C_10_H_14_O_4_
17.3	430.1481	429.1403	[M − H]^−^		41.559	Unidentified^a^	C_19_ H_26_O_11_
14.6	270.1109	269.1033	[M − H]^−^		40.696	Unidentified^a^	C_13_ H_18_O_6_
13.9	444.1268	443.1197	[M − H]^−^		34.745	Unidentified^a^	C_19_ H_24_O_12_
14.4	256.1310	255.1238	[M − H]^−^		30.969	1-Methoxy-1-(2,4,5-trimethoxyphenyl)-2-propanol^a^	C_13_ H_20_O_5_
11.6	216.0998	215.0926	[M − H]^−^		30.047	Unidentified^a^	C_10_H_16_O_5_

a METLIN formula database.

b CFM-ID.

c METLIN MSMS spectrum match.

d Authentic standard.

e Level I identification.

f Level II identification.

g Level IV identification. For the molecule features without identification level indicated the formula and identification from single MS library search.

### 3.2 Confirmation of identified biomarkers of broccoli intake

In order to confirm a dose–response for the putative biomarkers, metabolites were examined in urine samples following consumption of different portions of broccoli. The final population consisted of 32 participants, 5 of which did not complete all 3 of the test dinners but have data available for at least 1 meal. The study population demographics of the Dose–response study are reported in Table 1. The participants’ mean age was 29 years old, with a mean BMI of 23.96 kg m^−2^.

**Fig. 3:**
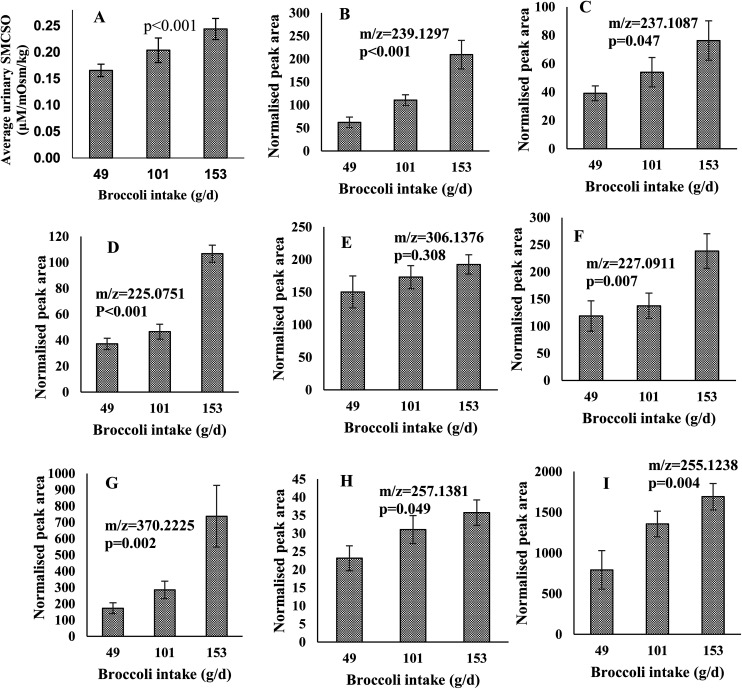
(A) Dose–response relationship between the fasting urine samples (mean and error bars (SEM) are presented) and amount of broccoli consumed. The *X*-axis, average broccoli intake (grams) during the Dose–response study; the *Y*-axis, SMCSO concentrations measured in urine (μM) normalised by osmolality (mOsm per kg). Repeated measure ANOVA was performed and SMCSO significantly increased responding to elevated broccoli intake in fasting urine samples (*p* < 0.001). (B–I) Dose–response relationship of the peak areas of fasting urine samples across the different portions of broccoli consumed. Values are means ± SEMs. The *X*-axis, average broccoli intake (grams per day) during the Dose–response study; the *Y*-axis, normalised peak area measured in urine normalised by osmolality (mOsm per kg). Figure titles indicated the *m*/*z* of features. The feature of *m*/*z* 255.1238 were identified in negative mode and the rest of features were identified in positive mode. One way ANOVA was performed to indicate the significant difference across three portions.

Urinary SMCSO concentrations were determined following intake of low (49 g), medium (101 g) and high (153 g) portions of broccoli. The average urinary SMCSO concentrations increased as broccoli intake increased (from 0.17 to 0.24 μM per mOsm per kg) (Fig. 3A). Repeated measures ANOVA indicated that SMCSO exhibits a dose–response relationship to broccoli intake in fasting urine samples (*p* < 0.001).

Features which exhibited an increased and differential time-course following broccoli consumption using LC-MS analysis were also examined for a dose–response relationship. Peak areas were examined for the 14 features of interest in positive and 7 features in negative following consumption of the different broccoli portions. Genipin (*m*/*z* = 227.0911), dihydro-β-tubaic acid (*m*/*z* = 237.1087), sinapic acid (*m*/*z* = 225.0751) and 5 unidentified compounds (*m*/*z* = 239.1297, 370.2225, 257.1381, 255.1238 and 306.1376) increased in normalised peak area as portion of broccoli increased (Fig. 3B–I). All were significantly increased with the exception of one feature (*m*/*z* = 306.1376).

### 3.3 Prediction of broccoli intake using a panel of biomarkers

One feature, SMSCO, from NMR analysis and 7 LC-MS features (*m*/*z* = 237.1087, 239.1297, 225.0751, 227.0911, 370.2225, 257.1381, and 255.1238) indicated a significant difference across the three portions and were selected into a panel of biomarkers. Using the training set approach a total of 11 out of 15 observations (73%) revealed good agreement between the predicted and actual intake for the low portion (Table 4). The agreement for medium portion intake and high portion intake were 80% and 67%, respectively (ESI Tables 1 and 2†).

**Table 4:** The predicted broccoli intake compared to actual broccoli intake by multimarker application (low portion intake)

Observation	Actual intake (g)	Predicted intake (g)	Standard deviation	2.5% percentile	97.5% percentile
1	**49**	**53.46**	24.84	30.66	110.96
2	**49**	**48.96**	13.68	28.58	93.52
3	49	91.56	26.64	37.42	124.37
4	**49**	**50.00**	18.37	29.91	104.23
5	**49**	**47.16**	10.27	25.22	65.4
6	**49**	**48.39**	11.06	28.81	73.02
7	**49**	100.73	15.75	59.14	136.24
8	**49**	**47.47**	9.65	25.45	64.85
9	**49**	**50.18**	14.27	31.81	98.83
10	**49**	**49.59**	11.26	32.19	49.59
11	**49**	100.39	18.15	49.32	100.39
12	49	94.99	22.01	42.12	94.99
13	**49**	**48.16**	10.46	28.57	68.79
14	**49**	**48.63**	10.26	29.42	69.18
15	**49**	**48.50**	11.22	29.22	75.83

## 4 Discussion

The current study confirmed SMCSO as an NMR-based urinary biomarker of broccoli intake and suggested several potential LC-MS-based biomarkers including genipin, dihydro-β-tubaic acid and sinapic acid. Importantly the work demonstrated a dose response relationship with increased urinary excretion as the amount consumed increased. Furthermore, a panel of biomarkers was capable of estimating broccoli intake using the multiMarker web application. Overall, the present study illustrates the application of metabolomic-based approaches for identifying food intake biomarkers and importantly highlight the potential of such biomarkers in determination of intake.

The panel of biomarkers were combined using the multiMarker model, a latent variable model, which allows prediction of food intake when only biomarker data are available. Using the data from the A-Diet study, the model was successfully applied to estimate the relationship between biomarkers and apple intake and to subsequently determine apple intake from biomarker data.^21^ In the current study, the biomarker panel performed excellently to identify low and medium levels of broccoli intake. For high level of intake the model had difficulties discriminating between 101 and 153 g day^−1^.

This study employed an untargeted metabolomic approach for the discovery of broccoli intake biomarkers identifying discriminating compounds between fasting and 4 hours postprandial metabolomic profiles. Agreeing with previous literature we identified SMCSO as a biomarker of brassica intake. SMCSO is an organosulfur non-protein amino acid found naturally in brassica.^23^ SMCSO, and 3 of its metabolites, were identified in the urine samples of 20 men following 14 day consumption of a high cruciferous vegetable diet (500 g day^−1^ of both broccoli and Brussel sprouts).^12^ These NMR-identified metabolites were able to differentiate urinary metabolomes after a high cruciferous diet from urinary metabolomes observed after a diet excluding cruciferous vegetables and alliums in the same participants. The present study demonstrated that urinary SMCSO was detectable after a smaller, more achievable intake of cruciferous vegetables, 135 g compared to the 500 g day^−1^ used by Edmands *et al.*,^12^ which supports the use of SMCSO when examining cruciferous vegetable intake after experimental and habitual intakes. Our study has added to the research area by confirming that urinary concentration of SMCSO demonstrated a dose–response relationship, increasing as broccoli intake increased.

From the LC-MS analysis multiple potential broccoli biomarkers were identified. The 3-hydroxysebacic acid is a derivative of 3-hydroxydicarboxylic. It is possible that this potential biomarker is reflective of metabolism of the plant polymers ingested in broccoli and warrant further investigation, however it is unlikely this compound is specific to broccoli intake. Another feature identified by LC-MS was genipin, a geniposide-derived aglycone present in fruit of Gardenia jasminoides. Genipin has multiple food industry applications such as a functional food ingredient, a natural blue colorant, and it is used as part of traditional Chinese medicine.^24^ Therefore, it is difficult to determine the exact dietary source of genipin identified in urine samples, however it did demonstrate a dose–response relationship with broccoli intake so future investigation is necessary to confirm this compound as a broccoli intake biomarker. LC-MS analysis also identified some flavonoid derivatives. Fruit and vegetables are natural sources of flavonoids.^25^ As flavonoid derivatives are commonly found in multiple fruit and vegetables, individually, they will not be specific broccoli biomarkers. However, the combination of these compounds into a multi-biomarker panel brings important information.

Even though the majority of previous research has focused on isothiocyanates and their association with brassica consumption, there are a number of limitations associated with verifying these organosulfur compounds as robust markers of intake.^12^ Glucosinolates are consumed intact but the bioavailability of the isothiocyanates is affected by food processing techniques, pH of stomach and intestines and microbial digestion.^26,27^ As isothiocyanates are highly reactive compounds they bind easily with macromolecules reducing bioavailability. This rapid interaction leads to a huge diversity of isothiocyanates differing structurally based on their side chain.^28^ This diversity can affect metabolism and lead to increased concentrations of different conjugated metabolic products excreted as well as the mercapturic acid of parent isothiocyanate. Many studies have limited their analyses to derivatives of specific mercapturates based on the structure of parent glucosinolate and isothiocyanate structures which only count for a proportion of metabolites of original isothiocyanate dose.^28^ Hence, there is a need to perform mechanistic studies to identify metabolites of structurally related compounds metabolised through separate pathways to get a full overview of potential cruciferous exposure markers.

A potential limitation of the research is that the discovery and dose–response studies were carried out only in the Republic of Ireland and therefore confirmation of these findings would be required in other ethnic populations. This study has multiple strengths associated: a range of different study designs were used to examine the biomarkers in different scenarios. Confirmation of a dose–response in the background of habitual diet is a noteworthy strength. To the best of our knowledge this is the first study to examine the dose–response relationship of urinary biomarkers across multiple levels of broccoli intake. Furthermore, the predictive ability of the panel of biomarkers was examined. Further research is needed into biomarkers of broccoli-intake in order to establish their qualitative or quantitative applications for higher quantities of intake. Further work is also needed to fully validate the biomarkers identified to meet the validation criteria previously outlined.^29^

The present study has illustrated the successful implementation of an untargeted metabolomics approach in search of dietary biomarkers of broccoli intake. Urinary SMCSO was identified as a potential intake biomarker of broccoli in NMR analysis and exhibited a dose–response relationship with increasing broccoli intake. Several LC-MS features, a number of which are flavonoid derivatives were identified and dose–response relationships revealed. For both NMR and LC-MS analysis, the panel of molecule features as putative broccoli biomarkers indicate their potential to predict the intake and pave the way forward for its application in supporting and improving objective measurement of broccoli intake. Future work should examine the use of the panel of biomarkers in a range of ethnic groups and in larger observational studies.

## Author contributions

A. E. M. conducted the discovery and dose–response studies, analyzed data, and prepared the manuscript. X. Y. acquired and analysed data and prepared the manuscript. C. C. acquired part of the metabolomics data. L. B. designed research, analysed data and prepared the manuscript. All authors read and accepted the final version of the manuscript.

## Conflicts of interest

The authors have no conflict of interest.

## Supplementary Material

FO-014-D2FO03988E-s001
