# Supplementary material for: Metabolomic based approach to identify biomarkers of broccoli intake
Source: Food Funct. 2023 Aug 29;14(18):8586–96. doi: 10.1039/d2fo03988e (PMC10508089; doi:10.1039/d2fo03988e)
Supplement: FO-014-D2FO03988E-s001 [file FO-014-D2FO03988E-s001.pdf]

Electronic Supplementary Material (ESI) for Food & Function.
This journal is © The Royal Society of Chemistry 2023

Supplementary Material

Metabolomic based approach to identify biomarkers of broccoli intake

Aoife E. McNamara[1,2,*], Xiaofei Yin[1,2,*], Cassandra Collins[1,2], Lorraine Brennan[1,2]

[1] UCD School of Agriculture and Food Science, Institute of Food and Health, University College Dublin, Belfield, Dublin 4, Ireland

[2] UCD Conway Institute of Biomolecular and Biomedical Research, University College Dublin, Dublin, Ireland

[*] These authors contributed equally.

Corresponding Author:
Professor Lorraine Brennan
UCD School of Agriculture and Food Science,
Institute of Food and Health,
University College Dublin, Belfield, Dublin 4, Ireland.

Email: lorraine.brennan@ucd.ie
Phone: 00 353 1 7166815

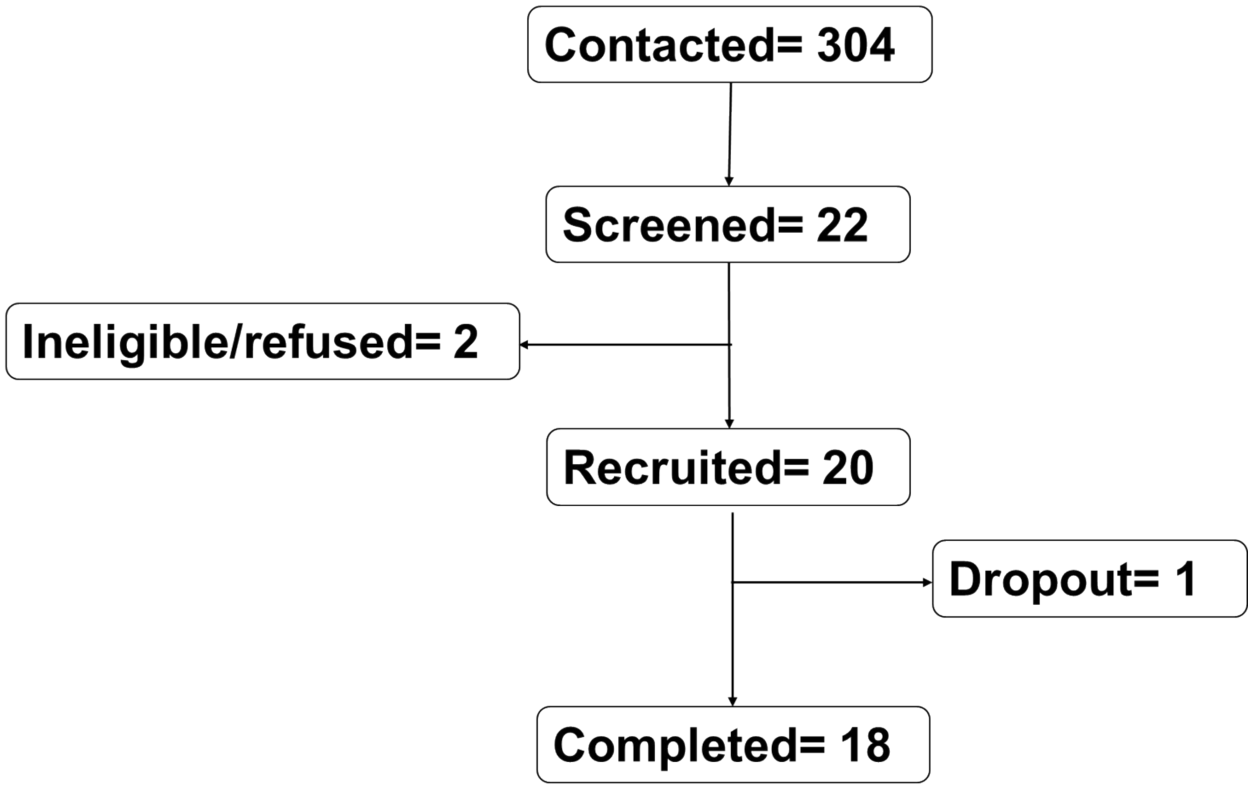

Supplementary Fig. 1 Flowchart of the study design. One participant attended baseline and a total of 18 completed the broccoli test meal.

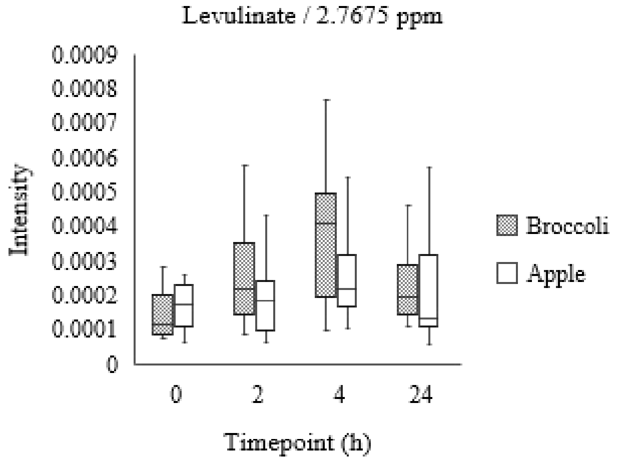
Levulinate / 2.7675 ppm
Intensity
0.0009
0.0008
0.0007
0.0006
0.0005
0.0004
0.0003
0.0002
0.0001
0
0
2
4
24
Timepoint (h)
Broccoli
Apple

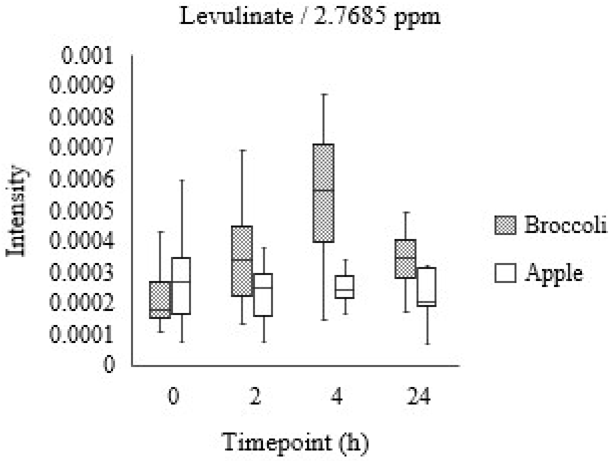
Levulinate / 2.7685 ppm
Intensity
0.001
0.0009
0.0008
0.0007
0.0006
0.0005
0.0004
0.0003
0.0002
0.0001
0
0
2
4
24
Timepoint (h)
Broccoli
Apple

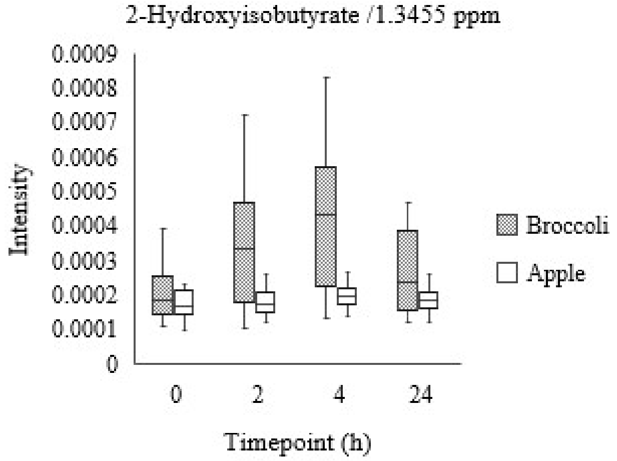
2-Hydroxyisobutyrate /1.3455 ppm
Intensity
0.0009
0.0008
0.0007
0.0006
0.0005
0.0004
0.0003
0.0002
0.0001
0
0
2
4
24
Timepoint (h)
Broccoli
Apple

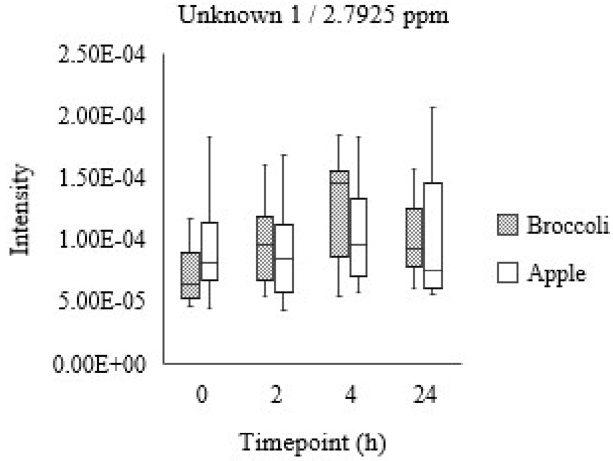
Unknown 1 / 2.7925 ppm
Intensity
2.50E-04
2.00E-04
1.50E-04
1.00E-04
5.00E-05
0.00E+00
0
2
4
24
Timepoint (h)
Broccoli
Apple

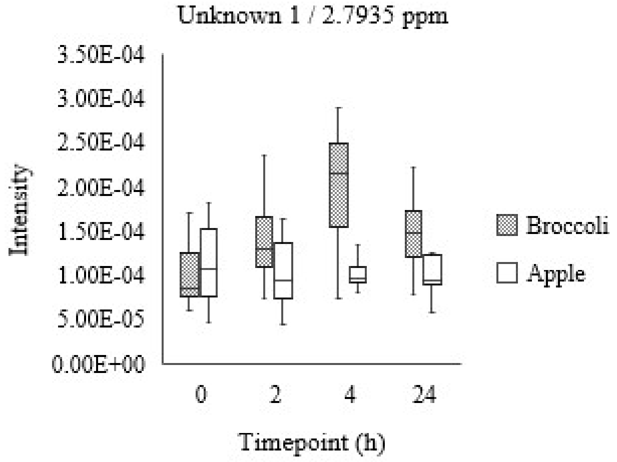
Unknown 1 / 2.7935 ppm
Intensity
3.50E-04
3.00E-04
2.50E-04
2.00E-04
1.50E-04
1.00E-04
5.00E-05
0.00E+00
0
2
4
24
Timepoint (h)
Broccoli
Apple

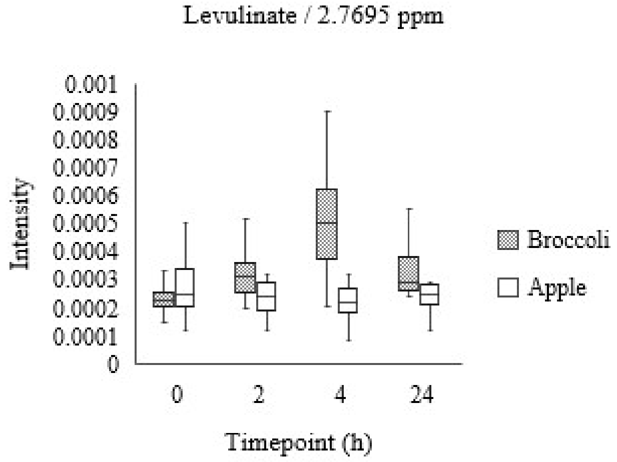
Levulinate / 2.7695 ppm
Intensity
0.001
0.0009
0.0008
0.0007
0.0006
0.0005
0.0004
0.0003
0.0002
0.0001
0
0
2
4
24
Timepoint (h)
Broccoli
Apple

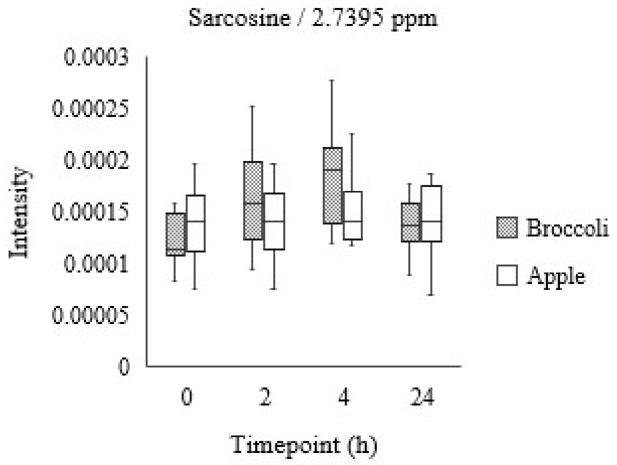
Sarcosine / 2.7395 ppm
Intensity
0.0003
0.00025
0.0002
0.00015
0.0001
0.00005
0
0
2
4
24
Timepoint (h)
Broccoli
Apple

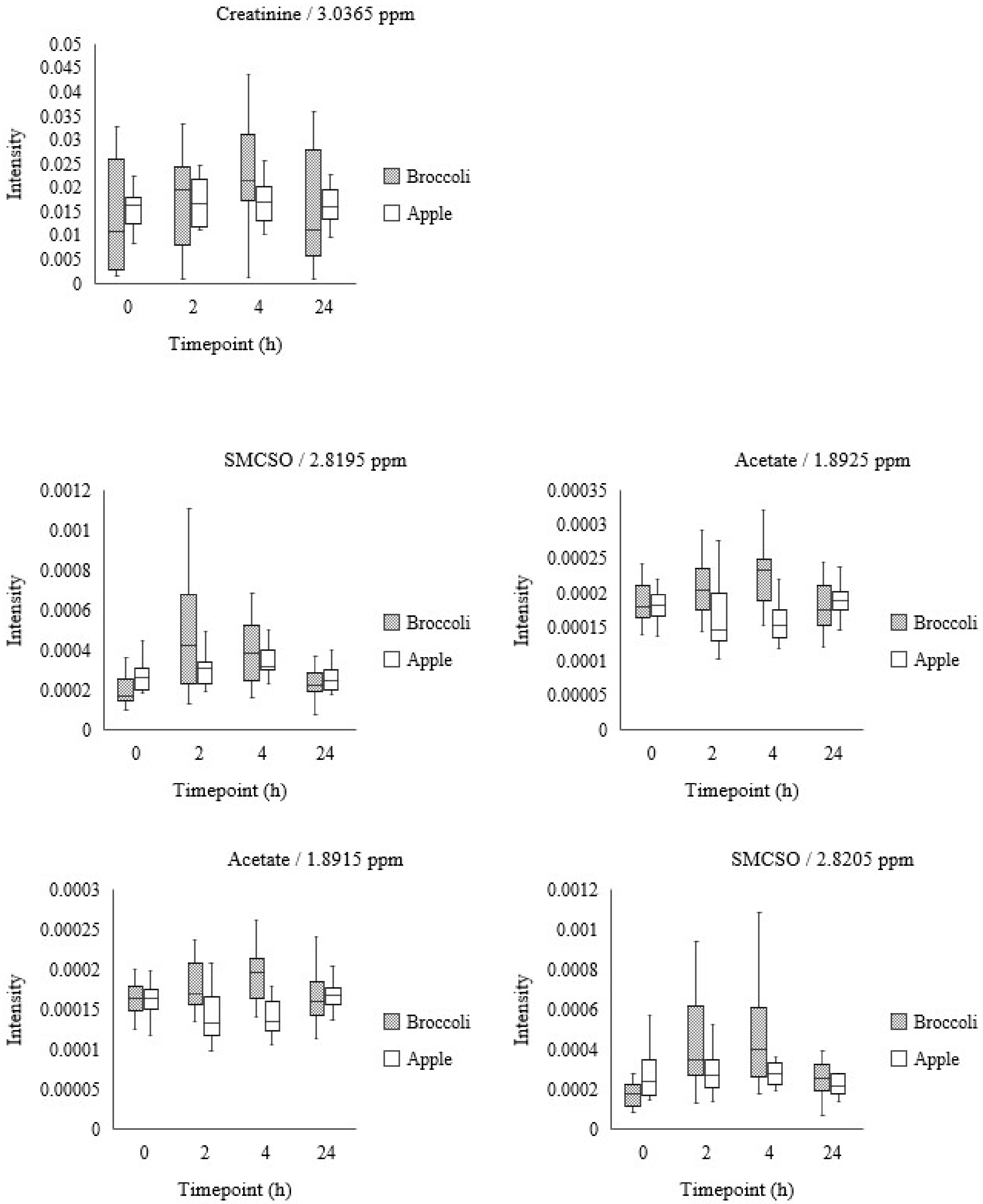

Supplementary Fig. 2 Box plots of the NMR spectral region intensities of potential urinary biomarkers over the 4 time-points (0, 2, 4, 24 h) following consumption of broccoli and apples. Grey and white boxes represent broccoli and apple group, respectively. The solid black line denotes the median of the group.

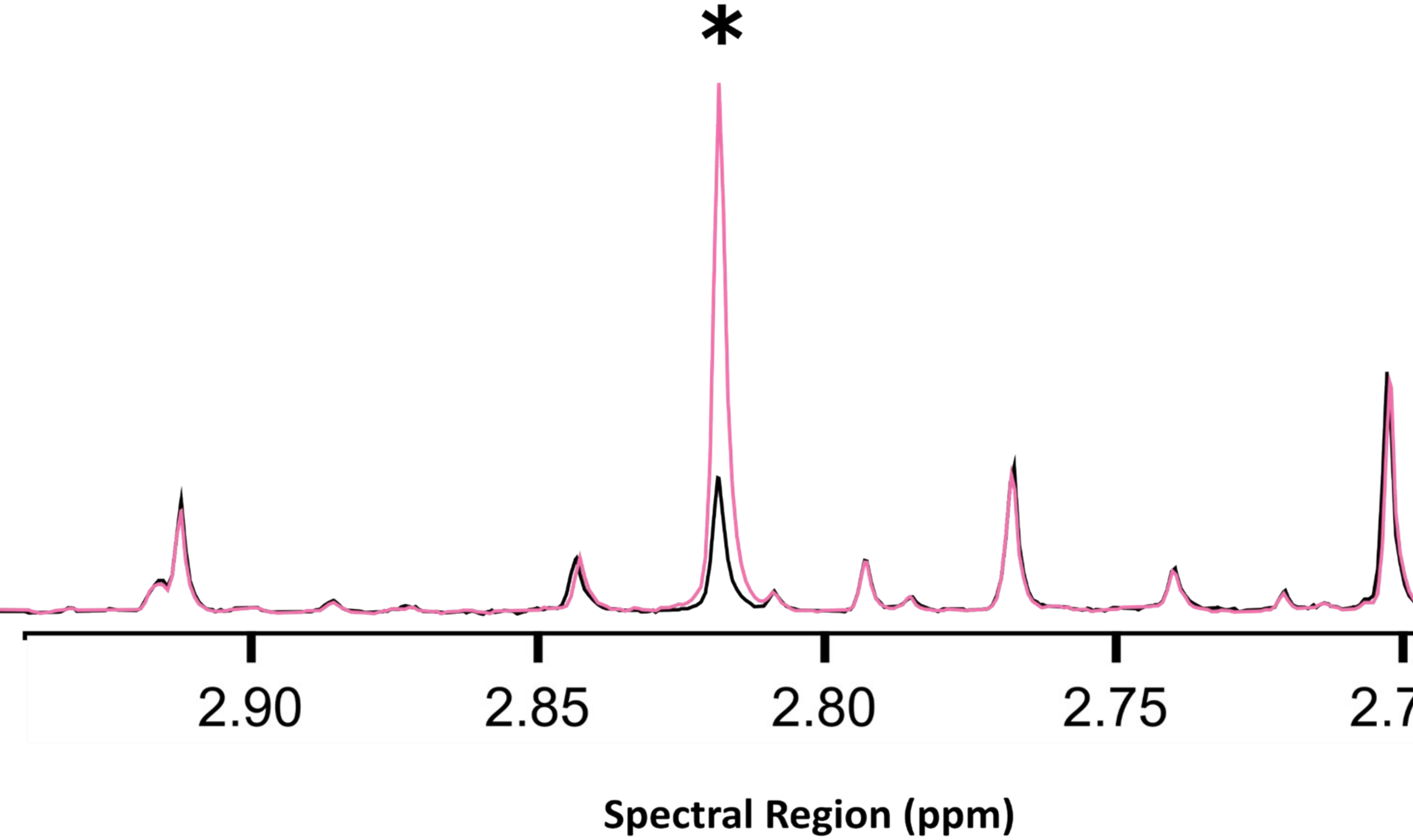

Supplementary Fig. 3 ^{1}H NMR spectrum of a urine sample to confirm the S-methyl cysteine sulfoxide (SMCSO) is correctly assigned. Black, urine sample; Red, urine sample spiked with standard of SMCSO. *indicated the spectral region (2.82ppm) of SMCSO.

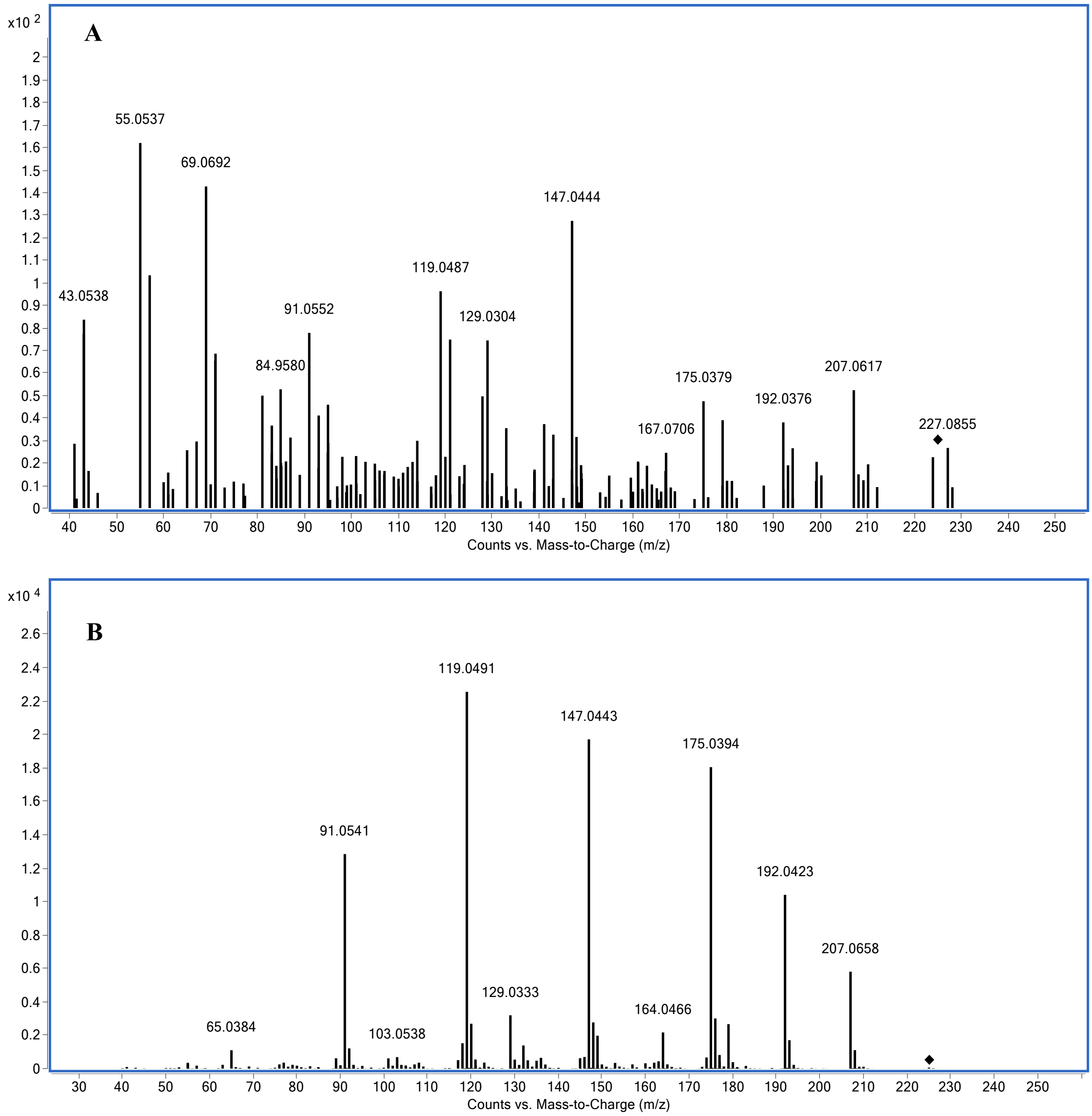

Supplementary Fig. 4 MS/MS spectra of Sinapic acid (225.0751 m/z, positive mode, 20 eV collision energy) in urine sample (A) and of a pure analytical standard (B).

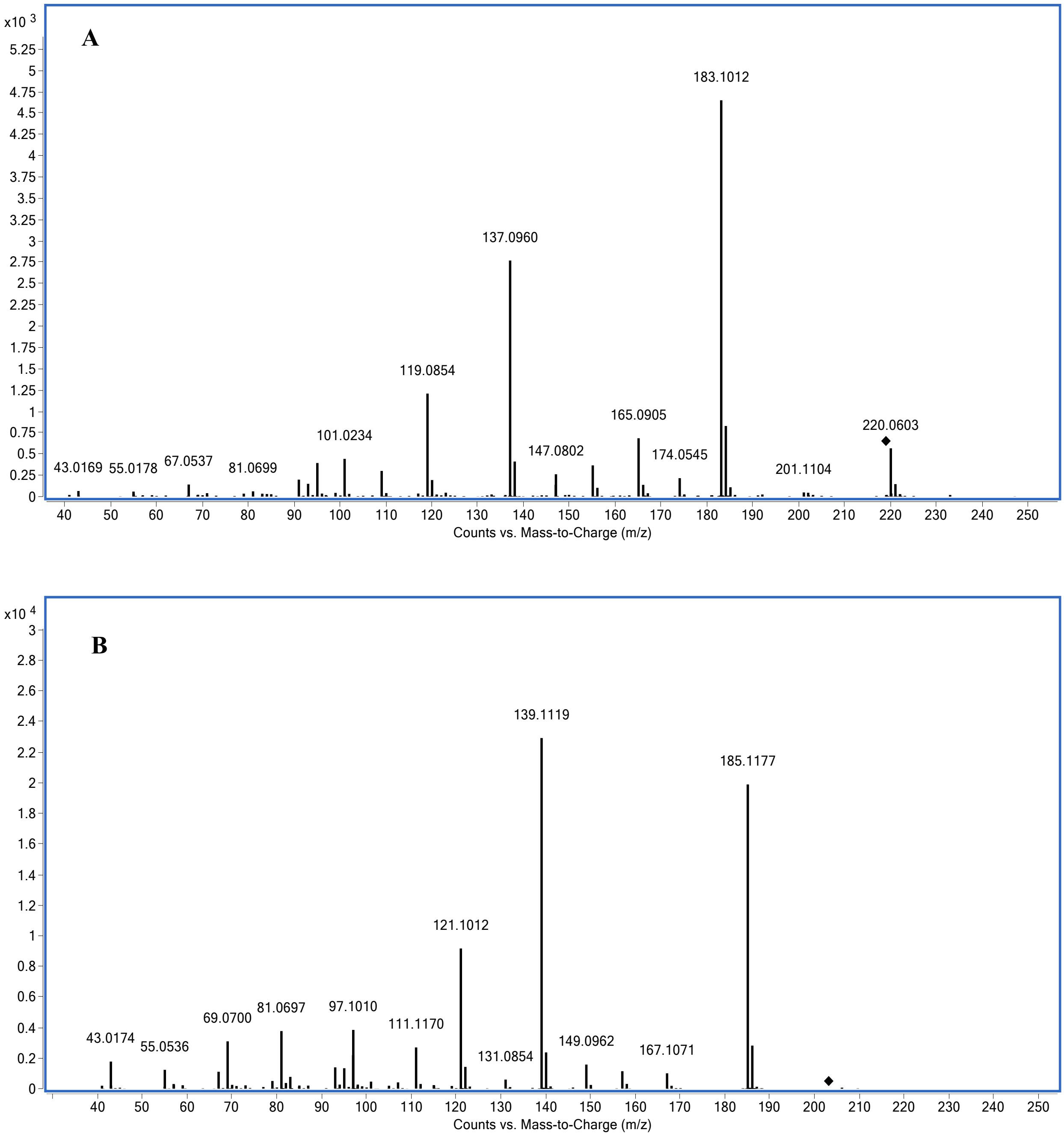

Supplementary Fig. 5 MS/MS Spectra of 3-hydroxysebacic acid (m/z 219.1206, positive mode, 10 eV collision energy) in urine sample (A) and MS/MS Spectra of a pure analytical standard of sebacic acid (m/z 203.1283, positive mode, 10 eV collision energy) (B). The 15.99 Da difference between the precursor of the urine spectra and standard can be contributed to the addition of a hydroxy group. The ~2 Da difference between the main

fragments (183.1012 Da vs 185.1177 Da, 137.0960 Da vs 139.1119 Da) can be explained by generating a double bond when losing the hydroxy group.

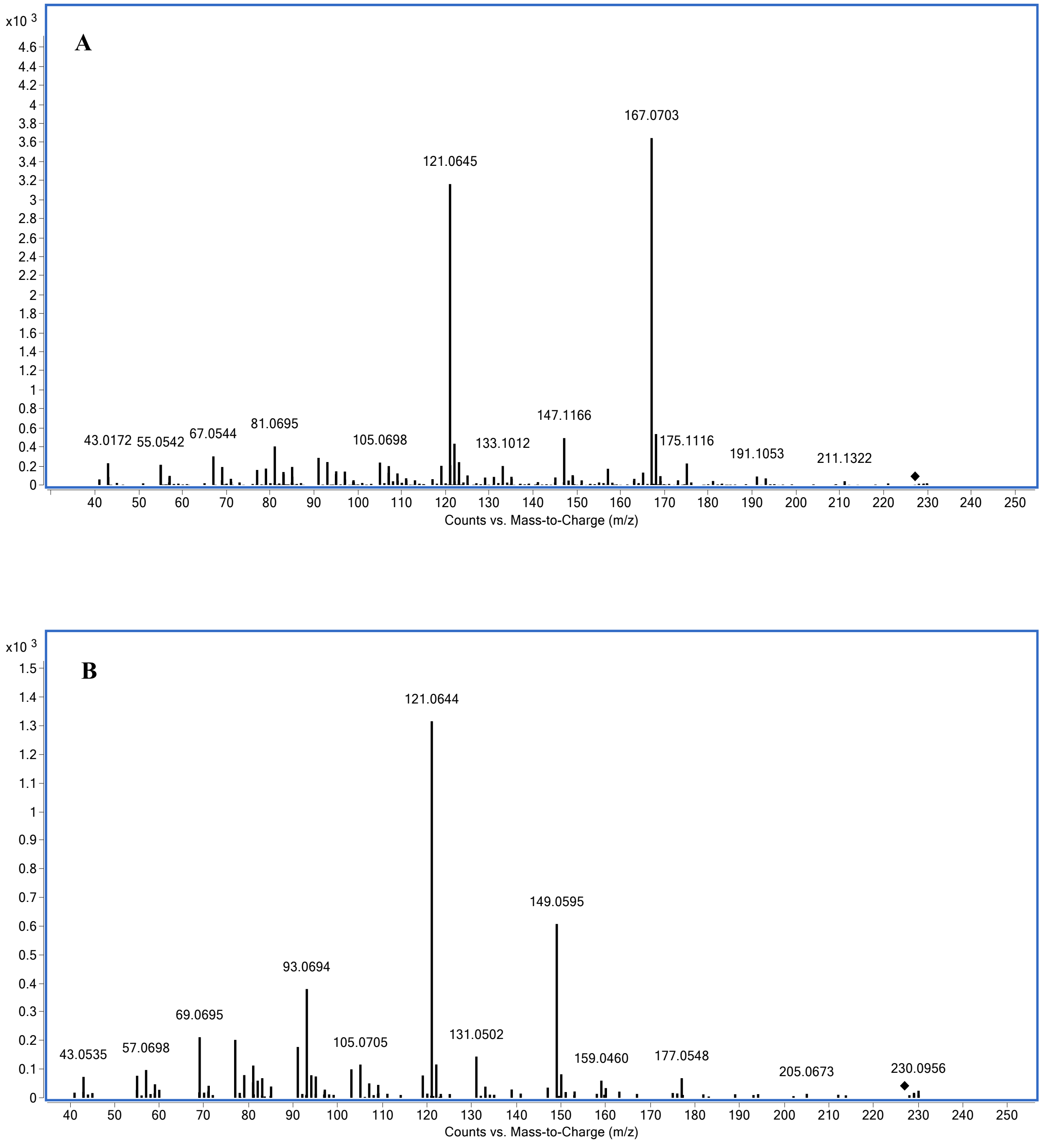

Supplementary Fig. 6 MS/MS Spectra of genipin (m/z 227.0912, positive mode, 20ev collision energy) in urine sample (A) and of a pure analytical standard (B). One of the main fragments in the standard was m/z 149.0595 however in the urine sample m/z 167.0706 was a prominent fragment, this may be due to a hydroxy group being kept in the urine sample.

Supplementary Table 1 the predicted broccoli intake compared to actual broccoli intake by multimarker application (medium portion intake)

Observation	Actual intake (g)	Predicted intake(g)	Standard deviation	2.5% percentile	97.5% percentile
1	**101**	**101.97**	22.2	50.78	157.36
2	**101**	**99.81**	22.93	45.57	151.6
3	**101**	**114.62**	30.87	50.57	171
4	**101**	**102.96**	19.65	59.88	155.46
5	101	64.66	26.2	35.51	115.06
6	**101**	**98.84**	19.59	45.64	127.54
7	**101**	**99.36**	17.05	48.11	121.24
8	**101**	**101.73**	18.19	61.05	148.82
9	101	**97.17**	21.15	42.89	118.5
10	**101**	**90.67**	24.28	39.1	90.67
11	**101**	**98.5**	18.03	47.57	98.5
12	101	51.59	18.41	34.19	51.59
13	101	**90.88**	26.12	38.88	120.47
14	**101**	**104.26**	26.02	50.9	161.3
15	**101**	148.66	21.21	95.04	177.86

The good agreement between actual and predicted intake was highlighted in bold.

Supplementary Table 2 the predicted broccoli intake compared to actual broccoli intake by multimarker application (high portion intake).

Observation	Actual intake (g)	Predicted intake(g)	Standard deviation	2.5% percentile	97.5% percentile
1	**153**	**156.93**	23.38	109.23	212.83
2	**153**	**160.16**	23.7	123.94	223.62
3	**153**	**166.91**	28.26	137.84	247.14
4	**153**	**156.23**	20.57	118.05	206.56
5	153	102.14	25.15	49.25	158.62
6	153	**163.27**	25.03	134.36	234.55
7	**153**	**150.27**	26.68	97.57	201.89
8	153	232.91	37.24	161.29	306.33
9	153	100.68	14.87	55.83	128.28
10	153	105.63	21.43	81.59	160.09
11	**153**	**155.41**	17.81	112.37	193.41
12	**153**	**135.28**	24.71	88.1	168.54
13	**153**	**158.64**	17.63	133.13	205.77
14	**153**	**152.41**	16.91	110.7	184.56
15	153	106.57	24.03	79.27	163.71

The good agreement between actual and predicted intake was highlighted in bold.
